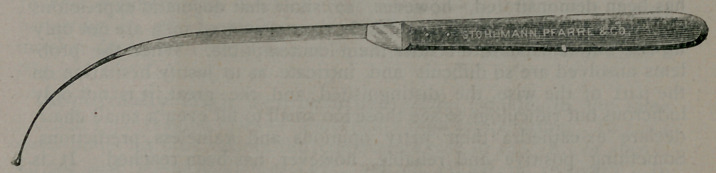# Abstracts and Gleanings

**Published:** 1881-01-20

**Authors:** 


**A New Knife for Fistula in Ano.**—Dr. Charles B. Kelsey, Surgeon to the Infirmary for Diseases of the Rectum, New York, in New York Medical Record, writes:

"In the operation for fistula, as ordinarily performed, the introduction of the director through the tract and bringing it out at the anus is the most painful step. The idea of the instrument represented in the accompanying cut was first suggested to me by seeing how often, in simple cases, the whole operation might be completed in an instant if the probe which is used to follow the tract in the first instance were only a knife, and if the director could be dispensed with; for, when once the probe is in the rectum, it may be brought out at the anus with little additional pain, and the parts are all ready to cut. With the instrument shown, it is often possible to operate without either, and with no pain except that from a rapid cut with a sharp knife. The figure shows the instrument half size. It is simply a strong curved bistoury with a light silver probe welded to its end. There should be no shoulder where the probe joins the cutting edge. The knife, it is evident, is applicable to the simple cases of the disease.

"NOTE.—Since this was written, my friend, Dr. J. L. Little, has called my attention to a plate in Heister's "Surgery," published in 1768, representing a much larger, sickle-shaped knife, but made on the same plan as this and intended for the same purpose. As the instrument seems to have been entirely discarded and forgotten, however, I have concluded to introduce it once more to the profession."

### ABSTRACTS AND GLEANINGS.

### THE MEDICAL YEAR, 1880.

We extract from an article in Medical Bi-Weekly, the following resume of medicine for the past year:

The Porro operation does not seem to have sustained the character it temporarily secured during the year 1879. Two-thirds of the cases in 1880 have died, fifteen out of twenty-two; while in 1879, out of seventeen cases, ten recovered. The mortality has therefore been greater in this operation in this country than in the Cæsarean operation; only twenty-six in sixty-two after the Porro operation have recovered; while in the Cæsarean operation there have been fifty recoveries in one hundred and twenty operations. Emmet's views and practice have gained ground, and his followers have certainly largely increased. Battey's operation is yet sub-judice. There have so far been one hundred and thirty well authenticated cases, by thirty-six operators, all men of distinction—eighty-six recovered and twenty-three died after laparotomy; seventeen recovered, and four died after elytrotomy; a mortality of nineteen per cent. for the vaginal section, and a fraction over twenty per cent. for the abdominal; a joint mortality of over twenty per cent. Such mortality makes this operation justifiable only after all other means of relief have failed. The future must determine more clearly the nature of the cases in which it should certainly be performed. There is nothing of especial interest to note further in this department. The best men are patiently at work, and their future statistics will indicate the truth.

In general surgery the chief attention of the most prominent operators has been given to the question of Listerism. Bryant, Spencer, Wells, Holmes, Lister, Sir James Paget, MacCormac and many others abroad and at home have debated this question in lectures and with the pen, but so far the results are not entirely satisfactory. Enough has been demonstrated, however, to show that dogmatic expressions on the part of obscure and relatively inexperienced men are not only unwise but calculated to render them contemptible. When the problems unsolved are so difficult and intricate as to justify hesitation on the part of the wise, the distinguished and the great, it is not only ludicrous but ridiculous to see those too small to fill even a small chair, declare ex-cathedra their petty opinions and valueless predictions. Something positive and reliable, however, has been reached. It is true that Runke and Klebs have proved that organisms develop about and underneath antiseptic dressings, but Cheyne has proved that there are two kinds of organisms principally, the bacteria and micrococci, and that they are not interchangeable. He has proved that while bacteria are always associated with putrefactive changes, the presence of micrococci is not indicative of putrefaction or decomposition. And that while micrococci which are not indicative of putrefaction may be found under antiseptic dressings, the bacteria, whose presence indicates always putrefactive changes are never found where antiseptics are freely used. The presence of micrococci after Listerian methods of dressing, therefore, does not prove any failure of antiseptics, for these organisms do not spring from putrefactive forces; but the bacteria organisms always to be seen where putrefaction occurs, are never seen when antiseptic dressings are efficiently prepared and adjusted. This is a positive advance. There is nothing else especially to be noticed in regard to general surgery in the past year.

Materia Medica has made no definite advances. Duboisia is slowly gaining its way as a mydriotic. Jaborandi, or its alkaloid, pilocarpine, is definitely accepted as a most valuable remedy in the inflammations of the serous membranes. Pisciatia crythrina, the Jamaica dogwood, has proved to be very efficient as an anæsthetic and hypnotic. The bromide of ethyl is generally now regarded as a dangerous agent for the production of anæsthesia. The many deaths occurring during the administration of sulphuric ether show that its much vaunted superiority over chloroform is not justifiable.

**New Respirator.**—We cull from Canada Lancet the following points of interest: In the field of general medicine there is nothing startling, though much that is interesting. Dr. Morell McKenzie has invented a respirator for the antiseptic treatment of phthisis pulmonalis. It covers both mouth and nose, and has a double breathing chamber for containing pieces of sponge saturated with a strong solution of carbolic acid or creasote. It is worn as continuously as possible, night and day. He does not claim that phthisis is cured by this plan, but that night-sweats, cough and impaired appetite are ameliorated. Picrotoxine has been used with success by Dr. Murrell in the treatment of night-sweats in phthisis. One drachm, of a 1 to 180 solution, is added to eight ounces of water, and a teaspoonful given at bed-time. Pilocarpine was also used by the same investigator, in doses of one-twentieth of a grain at bed-time, with beneficial results. After the sweating is checked by this remedy it does not return for several weeks.

**Fluid Extract of Ergot.**—Dr. Jones, in the British Medical Journal, reports a case in which copious hemorrhage from the lungs, occurring in pneumonia, was arrested by fluid extract of ergot, in drachm doses, with one ounce of liquor ammonia acetatis, four times a day.

**Abnormally High Temperature.**—Dr. Donkin, in the same journal, reports some cases of abnormally high temperature. In eight cases under his observation, the temperature rose to 108° F., or above. In one case it was as high as 117°, yet all ended in recovery. In some a rapid fall took place, in others there was considerable sweating with the high temperature.

**Desiccated Defibrinated Blood.**—Dr. J. W. Teale also reports a case of rheumatic fever in a female in which the temperature reached 117° F. The use of desiccated defibrinated blood as an agent especially adapted for rectal alimentation, has been brought prominently forward during the past year. The blood thus prepared contains all the elements of blood, except water and fibrine, and is soluble in water below 160° F. A drachm of the dried specimen represents an ounce of ordinary blood, and the quantity to be used in the course of twenty-four hours is from four to six ounces.

**Alkalies in Anemia.**—The use of alkalies in anemia has been brought forward by Dr. Nicholson, in an interesting article in the Practitioner. His theory is that anemia is frequently produced by hepatic disorder; that hepatic anem'a is one of the most common forms, and that as alkalies, especially potash, have a beneficial action on the liver and tend to restore the blood to its normal character, they should be administered in place of iron in the treatment of anemia.

**Chronic Dysentery and Diarrhœa.**—Bichloride of mercury in minute doses has been found particularly valuable by Dr. Reed, (Medical Times, Philadelphia,) in chronic dysentery and diarrhœa. He gives several cases successfully treated by this remedy. Dr. Ralfe (Lancet), on the other hand, gives his expeiience of the management of chronic dysentery by the castor oil treatment. Bismuth hæmatoxylon and turpentine were also used in addition to the oil in some of the cases. He also lays great stress upon rest and strict attention to diet, as essentials to the cure of this disease.

**Camphor and Chloral Hydrate,**—Equal parts, have been successfully used to quiet unruly and sleepless patients, by Dr. Simmons (American Journal of Medical Science). In cases of violent mania, delirium tremens, etc., he has found the mixture capable of accomplishing what other sedatives failed to do. In doses of twenty grains, it will produce effects which are altogeter beyond the reach of twenty grains of either camphor or chloral hydrate, in the same dose, to accomplish when administered alone.

**Tonga.**—Dr. Sidney Ringer, who has investigated the new remedy called Tonga, in use among the Fiji islanders, says that the fluid extract, in drachm doses, cured promptly six cases of neuralgia, improved the seventh, and failed in the eigthth, only because the preparation had become inert. Large doses, as half an ounce, produced slight drowsiness in one patient.

**Carbonate of Ammonia.**—Dr. J. P. Thomas, in Virginia Medical Monthly, strongly urges the use of carbonate of ammonia in diseases of the respiratory system, and especially in pneumonia. His theory of its action is, that it prevents the accumulation of carbolic acid in the blood, by promoting oxygenation. It also renders the blood alkaline and checks exudation. He administers it in doses of twenty to fifty grains. He considers it a certain prophylactic in heart clot, and says that it has often prevented death from this cause in pneumonia.

**Treatment of Lead Colic by Electricity.**—A case of the successful treatment of lead colic by electricity is recorded by Rothe, in Memorabil. There was obstinate constipation, which strong purgatives failed to overcome. The negative pole of a Faradic battery was inserted in the rectum, and the positive pole over the abdomen, and a strong current allowed to pass for eight or ten minutes. Very soon after a copious evacuation of the bowels occurred, followed by amelioration of the symptoms and recovery.

**"Peritoneal Surg‸ry."**—In reference to "peritoneal surgery" it is now the general impression among surgeons that in the present state of our knowledge and experience, exploration of the abdomen should be reserved for the most intractable cases of acute intestinal obstruction, the mortality so far not being less than fifty per cent. As confidence is gained in our means of diagnosis and treatment many patients that are now lost by delay may be saved. In chronic obstruction from cancer, tumors, etc., the utility of surgical interference is sufficiently proved by the results, and laparotomy, enterotomy or colotomy, may be had recourse to in suitable cases with a warrantable prospect of success. Rapid lithotrity with Biglow's improved aspirator has been fully endorsed by Sir Henry Thompson, R. T. Weir, and others. In cases in which difficulty has been encountered in removing the last few fragments, it is recommended to leave them to a future sitting rather than greatly to prolong the operation with a view to their immediate removal. This new procedure has, to a considerable extent, diminished the number of cases of lithotomy. Dr. Weir issues the injunction at the conclusion of his paper that only those who have had experience in lithotrity or who have made themselves familiar, on the cadaver, with this instrumental manipulator, should undertake the operation.

**Intra-Uterine.**—The subject of intra-uterine medication is still under discussion. Dr. Atthill, the great apostle of the frequent use of this method of treatment, still adheres to his practice, but has now come to regard carbolic acid as the safest and generally the most efficient agent. He uses a mixture of two parts acid to one of spirit or glycerine. He also speaks favorably of iodized phenol (iodine and carbolic acid), especially in endometritis occurring in old women. Some deaths have been reported from intra-uterine injections of perchloride of iron, one by Drs. Herman and Brown, in Obstet. Journal, Great Britain. The strength used was one to six, and the fluid was injected by a Higginson's syringe, to which a long uterine tube was attached. After a few syringefuls had been thrown up, the patient gave a faint cry, threw up her arms, turned pale, gasped for breath, and after a few inspirations died. A thrombus formed in the uterine veins and carried to the heart, was supposed to have caused the fatal result.

**Venesection.**—From the tone of the papers read at the different societies, and articles in the Journals of the "lost art" of venesection, it would appear that the practice is about to be revived, especially in the treatment of pneumonia. The abstraction of blood, is by most writers at present, regarded as of paramount importance to relieve engorged vessels, and prevent the effusions which always render the disease a grave one.

**Jamaica Dogwood,**—The use of Jamaica dogwood as a substitute for opium, has been highly recommended by those who have investigated its properties. It is more decidedly hypnotic than opium, produces no anorexia headache, and does not constipate the bowels or interfere with digestion. It acts rapidly, but its effect is less durable than opium, and requires to be given more frequently. The dose is twenty minims of the fluid extract every three hours.

**Treatment of Cancer by Chian Turpentine.**—Great prominence was given during the early part of the year to the treatment of cancer of the uterus by chian turpentine, brought forward by Prof. Clay, of Manchester, England. The remedy proved efficacious in his hands, and in the practice of a few others, but it has not been so generally efficacious in its effects as to warrant the high hopes at first entertained regarding its use. It seems, however, in most cases, to arrest the progress of the disease, and relieve pain, and, if given sufficiently early, may, in some cases, prove an effectual cure. It is best given in emulsion. One ounce of chian turpentine is dissolved in two ounces of pure sulphuric ether; to half an ounce of this ethereal solution, add four ounces of solution of tragacanth, one ounce of syrup, and enough water to make a sixteen-ounce mixture; the dose is two tablespoonfuls three times a day.

**Otology.**—One of the best papers of the year in otology is by Dr. Theobold, of Baltimore, on "The Scepticism Prevalent Regarding the Efficacy of Aural Therapeutics." He shows very ably the great progress, both in diagnosis and treatment, which has been made during the past few years; how little justifiable, by facts, are the assertions that ear-ache cannot be relieved except by bursting of the drum-head; that it is dangerous to arrest a chronic purulent discharge from the ear; that people grow out of otitis chronica, and that perforation of the drum necessarily leads to permanent deafness.

**Boracic Acid in Surgery.**—The use of boracic acid in surgery, has shown it to be a drug of greater power and wider range of applicability, than was formerly supposed. It is used with success on old sores and ulcers, both simple and specific, and also in the treatment of large suppurating wounds and abscesses it has been found of espacial value. In ozena and otorrhœa it acts as a prompt deodorizer and alterative, lessens the discharge and promotes healthy action. As a lotion in chronic cystitis and chronic inflammation of mucous membranes in general, it has a decidedly beneficial action.

**Introduction of Tracheal Tubes by the Mouth.**—Dr. McEwen, of Glasgow, advocates the introduction of tracheal tubes by the mouth instead of performing tracheotomy, and gives several cases in which he has adopted this method with good results. He recommends their use not only in chronic but also in acute affections, such as œdema glottidis, etc. The respirations are carried on perfectly through them, the sputa expelled, and the deglutition effected while the tube is in situ.

**A New Antiseptic.**—A new antiseptic and anti-neuralgic has been brought under notice during the past year, named menthol, a crystaline solid derived from oil of peppermint. In some respects it resembles thymol. It destroys bacteria, and applied externally, relieves neuralgic pains.

**Quinine with the Bromides.**—Dr. Gray, in Archives of Medicine, gives his experience of the use of quinine, as increasing the sedative effect of the bromides, belladonna, hyoscyamus, etc. He thinks it also relieves the depression which these medicines usually produce.

**Salicylate of Calcium.**—The salicylate of calcium in the serous diarrhœa of infants has been highly extolled during the past summer, by Dr. Hutchins, of Brooklyn. He treated successfully twenty-seven cases, from two months to two and a half years of age, with this remedy alone. Other forms of diarrhœa, lienteric or inflammatory, required additional treatment. The dose was from two to five grains.

**Ergot in Diabetes Mellitus.**—The use of ergot in diabetes mellitus has been brought forward by Dr. Hunt, in the Practitioner. Dr. Pepper, of Philadelphia, was the first to suggest this treatment. The dose is one drachm of the fluid extract three times a day. The rationale of its action is not known, but it is supposed to act in some way upon the vaso-motor system.

**Sponge Tents.**—Dilatation of the cervical canal by sponge tents, laminaria, or tupelo, is now being more or less generally discarded, owing to the danger of sepsis, and either rapid dilatation or division of the canal bilaterally up to the vaginal junction used instead, where necessary.

---

**Pilocarpin.**—This, as is well known, is an alkaloid obtained from the leaves of jaborandi. It has already been referred to in the Reporter as exhibited in uræmia and albuminuria (July 3d, 1880). Dr. L. Von Hoffer, of Austria, has seen marked improvement in diabetes from hypodermic injection of one-third of a grain of the alkaloid.

Prof. Pick, of Prague, has given one-sixth of a grain of the muriate, once or twice a day, an hour after eating, and found it of some benefit in prurigo, pruritus and chronic urticaria; of little or none in eczema and psoriasis. A singular fact he noticed was its remarkable effect on the hair. It hastens recovery in alopecia areata, and acts even more favorably on seborrhœa; indeed, in most cases he says that continued use of pilocarpin exerted an important influence on the oiliness of the hair and on its growth. The skin becomes softer, more pliant and satiny; comedos and papules of lichen can be more easily pressed out or got rid of, the scurfiness of the scalp becomes less or disappears, the hair is less brittle, the new growth of lanugo hairs changes more rapidly into dense, properly pigmented ones. Under employment of the drug for months the general condition of the patient was not impaired; indeed, the appetite improved, and he was better nourished. (*Vierteljahrschift fur Dermatologie*, 1, 2880).

The drug also exerts a stimulating influence on the retina. Dr. Mecklenburg, in Berlin, Klin. Woch., No. 44, 1880, gives this case:

A strong and healthy male prisoner, twenty-four years old, who had never previously suffered with his eyes, suddenly became night blind; as soon as dusk set in he could see nothing. It was a case of hemaralopia. The pupils were greatly enlarged, but nothing else abnormal about the eyes.

After the usual means had been tried, Dr. M. injected subcutaneously—

| | | |
|---|---|---|
| ℞ | Pilocarpin muriat, | 0.1 |
| | Aquæ destil, | 5.0 |

The improvement was immediate, and after the third injection the patient was well.

In Berlin it has also been tried in syphilis, when it has reached its constitutional stage, principally by Dr. Lewin, of La Charite Hospital The following extract from the London Medical Press and Circular gives its advantages and disadvantages thus—

In the course of three years and a half he has treated thirty-two patients. Seventy-eight per cent. of the patients were cured. Of seven cases two were of serous form, and had resisted energetic mercurial treatment; the cure was incomplete, and it was necessary to have recourse to injections of corrosive sublimate to complete it. In five other cases the treatment had to be suspended on account of intercurrent complications (endocarditis, hæmoptysis, collapse).

The mean duration of the treatment was eighty-two days. The dose injected each time was usually fifteen milligrams. The cure would be shorter if the patients would have daily injections; but as soon as amendment of the symptoms begins they require less and less frequent applications of the remedy.

Pilocarpin seems to prevent relapses with greater surety than mercury or vegetable depuratives. But in respect to facility of application, certainty of result and rapid cure, this medication is inferior to injections of corrosive sublimate, and often leaves behind it extreme sensibility to the influences of temperature, which obliges patients, after the cure, to keep their room for some time, for fear of arthritic and rheumatic troubles.

In diphtheria it was editorially recommended in the Reporter, (vol. xliii, pp. 524, 540), on the strength of the assertion by Dr. Guttman, of Berlin. His prescriptions were, however, not quoted. They are as follows, he combining pepsin with the alkaloid, in order to combat the gastric catarrh present—

| | | | |
|---|---|---|---|
| ℞ | Pilocarpin muriat., | gm. 0.02—0.04 | |
| | Pepsin, | gm. 0.6 —0.8 | |
| | Acidi hydrochlor, | gtt. ij | |
| | Aquæ dest., | gm. 80.0 | M. |

Sig.—A teaspoonful hourly for children.

For adults—

| | | | |
|---|---|---|---|
| ℞ | Pilocarpin muriat., | gm. 0.03—0.05 | |
| | Pepsin, | gm. 2.0 | |
| | Acidi hydrochlor, | gtt. iij | |
| | Aquæ dest., | gm. 240 0 | M. |

Sig—Hourly, a tablespoonful.

Dr. Rothe, of Altenburg, in Med. Cent. Zeitung, Nov. 6th, says, that four years ago he tried jaborandi in two cases, but both died, and he renounced the experiment. This throws some doubt on Guttman's discovery.

The price of pilocarpin is high; it sells in the eastern cities at thirty-five cents per grain, which makes it almost prohibitive in many cases.

**Rheumatism.**—Dr. Evans, in Medical and Surgical Reporter, says: I wish to call attention to the use of large doses of quinine in the treatment of acute rheumatism. Lately I had a case in which the temperature was 110°, pulse 140. Large doses of quinine were used, and the patient recovered.

**For Chronic Rheumatism.**—Prof. Pepper, in Chicago Medical Journal, says: I have thus far made scarcely any allusion to the large group of valuable remedies—mostly of an alterative character—that have acquired reputation in the treatment of chronic rheumatism.

It is true that in no case of this kind can we afford to depend solely on the use of any of these remedies, to the exclusion of baths, massage, diet, hygiene, it is no less true that in nearly every case there are indications that call for the use of some one or more of them. It would be impossible to discuss at length the merits of the very numerous remedies of this class, so that I must limit myself to the bare mention of those which have proved most valuable in my own experience.

In cases of chronic rhenmatism limited to one or a few joints with considerable effusion, I have used the following with advantage:

℞ Potassi iodidi.................................... ʒ ij.

Hydrargyri bichloridi,................................ gr. j.

Syrup sarsæ comp ,.................................. ℥ v.

Ft. sol. S. Teaspoonful in water after meals.

or:

℞ Hydrargyri bichloridi,............................ gr. j.

Inf. gentianæ comp................................ ℥ vij.

Ft. sol. S. 1 to 2 teaspoonfuls in water after meals three times daily.

In cases where a number of joints are involved with marked tendency to exacerbations, and especially if the lesions of the small joints indicate gouty complications:

℞ Pulv. guaiaci,............................ ʒ j.

Vin colchici radicis,...................... ʒ ij to ʒ iij.

Potassii iodidi,............................ ʒ j.

Pulv acaciæ,................................ q. s.

Sp. lavendulæ comp......................... ℥ ss.

Aq. cinnamoni,............................. q. s. ad ℥ vj.

Ft. sol. S. Dessertspoonful three times daily in water.

The bicarbonate or the acetate of potash may often be substituted with advantage to the digestion for the iodide of potassium in the above mixture. I have already alluded to the use of prolonged courses of lithia as being very beneficial, especially in cases with a gouty element and with defective action of the kidneys. In regard to the mode of its administration, I much prefer the effervescing granulated salts.

I must also mention the benefit I have derived from the prolonged use of carefully increased doses of Donovan's solution. It is to be remembered that these alteratives have, for the most part, been given while the patient was also taking iron in large doses, cod liver oil, syr. hypophos. comp., or some similar nutriment.

I will merely mention the nitrate of silver as an alterative, from which I think I have obtained good results, especially in cases attended with neuritis and with marked nervous symptoms.

**Traumatic Tetanus.**—Dr. Morton, in Medical and Surgical Reporter, reports success in a case of traumatic tetanus with the following: Morphia was employed hypodermically, and conia in one-fourth drop doses, gradually increased to one-half drop, was given every two hours. Under this treatment he gradually but very slowly improved, until now he is able to be out of bed.

**Atrophy of Infants.**—In a case of wasting or dropsy in a child deprived of the breast: "The nursing-bottle was examined. The glass tube which extended to the bottom of the bottle was lined with curd, and a quantity of milk remaining from a supply placed in the bottle about an hour before was sour and contained numerous small curds. This change, it was stated, often occurred, in spite of much care taken to keep the bottle and tubing clean.

Directions were given to substitute a soft india-rubber nipple for the tubing, to keep both the bottle and nipple thoroughly clean, to wash out the child's mouth with cold water after each feeding, and to use a food composed of one part of barley-water to two of milk, with the addition of a tablespoonful of lime-water to each half-pint. Small doses of bicarbonate of sodium, with peppermint-water, were prescribed every three hours. The nurse was also ordered to rub half a teaspoonful of warm olive oil into the skin of the abdomen twice daily, to anoint the surface involved in the intertrigo with oxide of zinc ointment, and to keep the feet warm by frictions with the hand.

The improvement under this treatment was rapid. On April 11, (the day of the last visit), his mouth was cool and free from thrush; there was little eructation; the bowels were natural; there were no more attacks of colic; the sleep was undisturbed; the child had begun to gain weight; and the intertrigo was very much better."—*Medical Times*.

**Coca in the Opium Habit.**—In response to inquiries as to the use of coca in the opium habit, we give the instructions of Dr. Palmer, as published in the Louisville Medical News, as follows:

"Coca is to be used as a *substitute* for the opium. It is, therefore, to be taken as freely as the cravings of the system for opium may demand—tablespoonful doses of the fluid extract several times a day, more or less, as needed. The 'break-off' is to be made at once and for all, and coca is the staff upon which the sufferer is to throw his whole weight."

The fluid extract, as prepared by Parke, Davis & Co., Detroit, is recommended as a good preparation.

**Nasal Catarrh.**—Dr. Hamill, in Medical and Surgical Reporter, says: A man for five years had suffered from nasal catarrh. Almost everything had been tried without benefit, when he was recommended to plug the nostrils alternately with cotton. He found great relief from this simple treatment, and I call attention to it, so that others may try it.

Dr. Cathell, says: I have tried it and found it beneficial. I got the idea from a little article going the rounds of the press. A German was the originator, and had used it in fifteen cases; all got well; average duration of treatment, twenty-one days. I have used it for about one year, and know of no case which has not been cured or greatly benefitted. It gives rest to the irritated membrane. I do not use it in ozæna.

## Figures and Tables

**Figure f1:**